# Pharmacokinetics, Pharmacodynamics, Tolerability, and Food Effect of Cenerimod, a Selective S1P_1_ Receptor Modulator in Healthy Subjects

**DOI:** 10.3390/ijms18122636

**Published:** 2017-12-06

**Authors:** Pierre-Eric Juif, Daniela Baldoni, Maribel Reyes, Darren Wilbraham, Salvatore Febbraro, Andrea Vaclavkova, Matthias Hoch, Jasper Dingemanse

**Affiliations:** 1 Department of Clinical Pharmacology, Idorsia Pharmaceuticals Ltd., Allschwil 4123, Switzerland; daniela.baldoni@idorsia.com (D.B.); maribel.reyes.centeno@gmail.com (M.R.); Matthias.Hoch@astrazeneca.com (M.H.); jasper.dingemanse@idorsia.com (J.D.); 2 Quintiles Drug Research Unit, London SE1 1YR, UK; wilbraham_darren@lilly.com; 3 Simbec Research Ltd., Merthyr Tydfil CF48 4DR, UK; febbras@doctors.org.uk; 4 Department of Global Drug Safety, Actelion Pharmaceuticals Ltd., Allschwil 4123, Switzerland; andrea.vaclavkova@actelion.com

**Keywords:** pharmacokinetics, pharmacodynamics, S1P_1_ receptor modulator, lymphocyte, tolerability, food effect

## Abstract

The pharmacokinetics, pharmacodynamics, tolerability, and food effect of cenerimod, a potent sphingosine-1-phosphate subtype 1 receptor modulator, were investigated in three sub-studies. Two double-blind, placebo-controlled, randomised studies in healthy male subjects were performed. Cenerimod was administered either as single dose (1, 3, 10 or 25 mg; Study 1) or once daily for 35 days (0.5, 1, 2 or 4 mg; Study 2). A two-period cross-over, open-label study was performed to assess the food effect (1 mg, Study 3). The pharmacokinetic profile of cenerimod was characterised by a *t*_max_ of 5.0–6.2 h. Terminal half-life after single and multiple doses ranged from 170 to 199 h and 283 to 539 h, respectively. Food had no relevant effect on the pharmacokinetics of cenerimod. A dose-dependent decrease in lymphocyte count was observed after initiation of cenerimod and reached a plateau (maximum change from baseline: −64%) after 20–23 days of treatment. Lymphocyte counts returned to baseline values at end-of-study examination. One serious adverse event of circulatory collapse (25 mg dose group, maximum tolerated dose: 10 mg) and adverse events of mild-to-moderate intensity were reported. Treatment initiation was associated with transient decreases in heart rate and blood pressure at doses >1 and ≥10 mg, respectively.

## 1. Introduction

Sphingosine-1-phosphate (S1P) is synthesised and secreted by many cell types. This phospholipid is a ligand of and binds to G-protein-coupled receptors (S1P_1_–S1P_5_) to modulate a wide range of physiological systems [1,2,3]. Repeated administration of S1P_1_ receptor (S1P_1_R) modulators triggers a sustained internalisation of this receptor and induces a long-lasting inhibition of the egress of lymphocytes from lymphoid organs [4]. This reduction in lymphocyte count in peripheral blood is considered a therapeutic approach to treat autoimmune diseases. In this respect, the non-selective S1P receptor modulator fingolimod (Gilenya^®^) is used to treat patients with relapsing multiple sclerosis [5] and selective S1P_1_R modulators have achieved proof-of-concept in multiple sclerosis [6] and psoriasis [7]. Whereas cardiodynamic (heart rate [HR] reduction, delay in atrio ventricular (AV) conduction) and pulmonary effects had initially been related to S1P_3_ agonism [8,9,10], these effects were also observed with selective modulation of S1P_1_R [11,12,13,14,15].

Cenerimod (ACT-334441) is a potent S1P_1_R modulator. In vitro and in vivo preclinical studies revealed that cenerimod is highly selective for the human S1P_1_R. Indeed, compared to S1P, cenerimod is 16-fold more potent on the S1P_1_R (EC_50_: 1 vs. 16 nM for cenerimod vs. S1P) and 2000-fold less active on the S1P_3_ receptor (EC_50_: 228 vs. 0.1 nM for cenerimod vs. S1P) [16]. In animal species and man, the binding of cenerimod to plasma proteins is >99.9%. In rats and dogs, single- and multiple-dose administration of cenerimod at doses >0.3 mg/kg induced a dose-dependent and reversible reduction of lymphocyte count. In a mouse model of multiple sclerosis (experimental autoimmune encephalomyelitis), preventive and therapeutic treatment with cenerimod (6 mg/kg/day) led to clinical and histological efficacy. In rats, a decrease in HR was observed at doses ≥0.3 mg/kg, this reduction was similar at doses of 1 and 3 mg/kg. The pharmacokinetic (PK) profile of cenerimod (0.1 mg/kg) revealed a rapid absorption and a terminal half-life (*t*_1/2_) of 12 and 7.5 h in male rats and dogs, respectively. Multiple-dose administration led to a greater than dose-proportional exposure in both rats and dogs between 3 and 10 mg/kg.

The aim of the studies reported here was to assess for the first time in humans the PK, pharmacodynamics (PD), food effect (only after single dose), safety, and tolerability of cenerimod as a single oral dose or once daily (o.d.) multiple oral doses for 35 days.

## 2. Results

### 2.1. Pharmacokinetics

**Figure 1:**
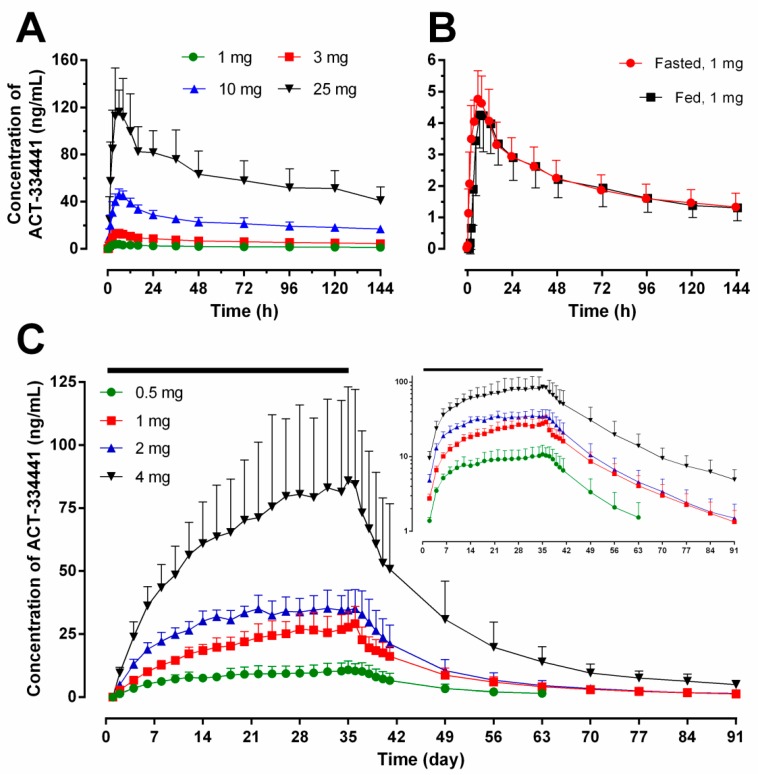
Arithmetic mean (with standard deviation) plasma concentration-time profiles of cenerimod following single or multiple o.d. oral doses in healthy subjects. Panels (**A**,**B**) represent the concentration of cenerimod after single ascending doses (*N* = 6 per dose group) and single-dose administration in fasted versus fed conditions (1 mg, *N* = 8), respectively; Panel (**C**) depicts the concentration of cenerimod until the end-of-study (EOS) (*N* = 6 per dose group, the black bar represents the treatment duration of 35 days) on linear scale (semi-logarithmic scale shown as inset).

As described in Table 1 and depicted in Figure 1A, after single-dose administration, cenerimod was slowly absorbed with a median time to reach maximum concentration (*t*_max_) between 5.0 and 6.2 h (range: 4.0–8.0 h). Cenerimod was eliminated from plasma with a geometric mean *t*_1/2_ (95% confidence interval (CI)) that varied from 170 (134–213) to 199 (188–211) h. Exposure to cenerimod was slightly more than dose proportional as estimated from the power model. The linear regression slope (95% CI) was 1.07 (1.00–1.13), 1.05 (0.99–1.11), and 1.07 (1.01–1.13) for maximum plasma concentration (*C*_max_), area under the plasma drug concentration-time curve (AUC) from 0 to 24 h (AUC_0–24_), and AUC from 0 to infinity (AUC_0–∞_), respectively.

In Study 2, similar *t*_max_ values were observed on Day 1 (5.0 to 6.0 h) and Day 35 (4.3 to 6.0 h). Cenerimod was eliminated from plasma with a geometric mean terminal *t*_1/2_ (95% CI) of 283 (210–381), 436 (359–529), 415 (358–481), and 539 (492–591) h following 0.5, 1, 2 and 4 mg doses, respectively. Cenerimod was shown to accumulate substantially over the 35 days of administration (Figure 1C), as reflected by higher *C*_max_ and AUC_0–24_ values on Day 35 than on Day 1 (Table 1). The accumulation ratio (geometric mean Day 35/Day 1 ratio) was 5–7 for *C*_max_ and 7–9 for AUC_0–24_ across the cenerimod dose groups (Table 1). Plasma concentration-time profiles of cenerimod over the 35-day dosing period revealed a gradual increase in trough concentrations and steady-state conditions at lower doses were reached after 20 to 32 days of treatment (Figure 1C). On Day 1 and Day 35, exposure to cenerimod was shown to be dose-proportional across all dose groups. The linear regression slope (95% CI) was 0.96 (0.84–1.07) and 0.93 (0.83–1.03) on Day 1 for *C*_max_ and AUC_0–24_, respectively, and 0.91 (0.75–1.08), 0.93 (0.75–1.10), and 1.00 (0.78–1.22) on Day 35 for *C*_max_, AUC_0–24_, and AUC_0–∞_, respectively.

Volume of distribution at steady-state (Vss/F) increased with dose. The geometric mean (95% CI) Vss/F was 758 (525–1095), 874 (592–1290), 1294 (1157–1446), and 1483 (947–2324) L at doses of 0.5, 1, 2 and 4 mg, respectively. Geometric mean (95% CI) clearance (CL/F) was comparable across dose groups, between 1.39 (1.08–1.79) and 2.16 (1.79–2.61) L/h.

The concentration-time profiles of cenerimod under fed or fasted conditions are depicted in Figure 1B. A slightly slower rate of absorption reflected by a higher median *t*_max_ (7.0 (min–max: 6.0–12.0) h) and a lower *C*_max_ (4.35 (3.5–5.4) ng/mL) was observed following cenerimod administration in fed condition compared to fasted condition (*t*_max_: 6.0 (6.0–8.0) h; *C*_max_: 4.73 (4.1–5.5) ng/mL). Table 1 reveals a similar exposure to cenerimod following fed and fasted administration. The geometric least squares (LS) mean ratio (90% CI) (fed (reference) vs. fasted, in %) was 92.0% (84.97–99.54), 90.2% (84.52–96.31), and 97.1% (87.55–107.69) for *C*_max_, AUC_0–24_, and AUC_0–∞_, respectively. The 90% CIs of the geometric LS mean ratios were all within the limits of 80–125%.

### 2.2. Pharmacodynamics

**Figure 2:**
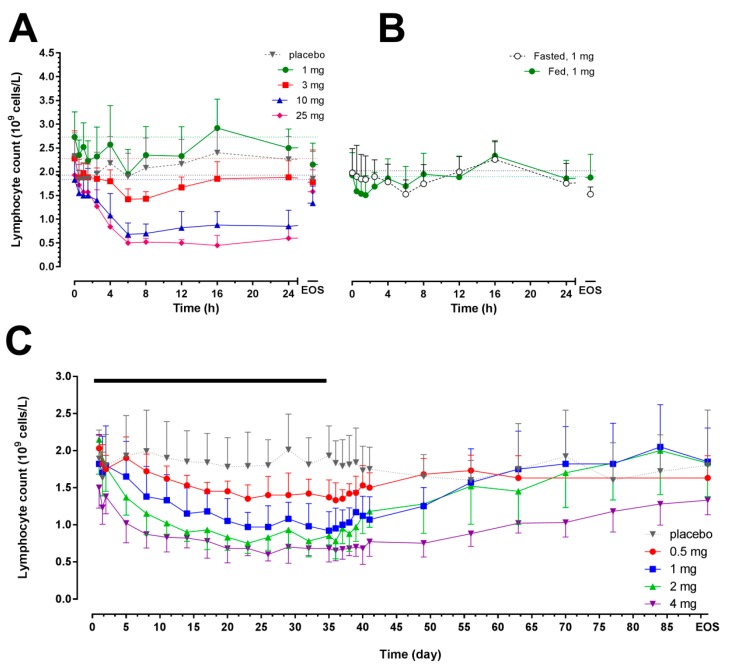
Arithmetic mean (with standard deviation) lymphocyte count in peripheral blood versus time profiles from 0 h to EOS after single-dose administration of cenerimod (Panel (**A**), *N* = 6 per dose group, *N* = 8 for placebo group); single-dose administration in fed or fasted conditions (Panel (**B**), *N* = 8); and after multiple o.d. oral doses for 35 days (Panel (**C**), *N* = 6 per dose group, *N* = 8 for placebo group). The black bar represents the treatment duration. The horizontal dotted lines represent the baseline values. EOS: end-of-study.

In Study 1, a dose-dependent decrease in lymphocyte count was observed following single-dose administration of cenerimod at doses ≥3 mg (Figure 2A). At doses ≥3 mg, a significant decrease in lymphocyte count was observed (*p* < 0.05 baseline vs. nadir in the 3, 10 and 25 mg dose groups) and the maximal mean percentage change from baseline (± standard deviation (SD)) was significantly more pronounced compared to placebo: −34.5 ± 13.8% (8 h post-dose, *p* = 0.01), −60.7 ± 11.4% (8 h post-dose, *p* < 0.0001), and −76.1 ± 10.4% (16 h post-dose, *p* < 0.0001) in the 3, 10 and 25 mg dose groups, respectively. This effect was also reflected by the maximum effect (*E*_max)_ and area under the effect curve (AUEC) values (Table 2).

In Study 2, when administered o.d. for 35 days, cenerimod led to a gradual, dose-dependent reduction in lymphocyte count that reached a plateau 20 to 23 days after first dose (Figure 2C, Table 2). The maximum (%) change from baseline (mean ± SD) was −33.6 ± 15.0% (Day 36, *p* = 0.0002 vs. baseline), −50.1 ± 4.4% (Day 35, *p* = 0.0009 vs. baseline), −64.1 ± 8.7% (Day 23, *p* < 0.0001 vs. baseline), and −56.3 ± 7.0% (Day 36, *p* < 0.0001 vs. baseline) following doses of 0.5, 1, 2 and 4 mg, respectively. The nadir lymphocyte count was observed in the 4 mg group (0.60 ± 0.09 × 10^9^ cells/L). In the placebo group, the largest decrease was −13.8 ± 12.9% (*p* = 0.22) and lymphocyte count was ≥1.0 × 10^9^ cells/L during the entire study. 

In Study 3, a similar maximal mean decrease in lymphocyte count was observed in fed (−20.5 ± 14.6%, 6 h post-dose; *p* = 0.049 vs. baseline) and fasted (−20.6 ± 12.1%, 1.5 h post-dose; *p* = 0.08 vs. baseline) conditions (Figure 2B). Food had no effect on the time to nadir, *E*_max_, and AUEC (Table 2).

Lymphocyte count in each subject returned to at least the lower limit of the normal range (i.e., 80% of baseline) at end-of-study.

### 2.3. Pharmacokinetics/Pharmacodynamics

**Figure 3:**
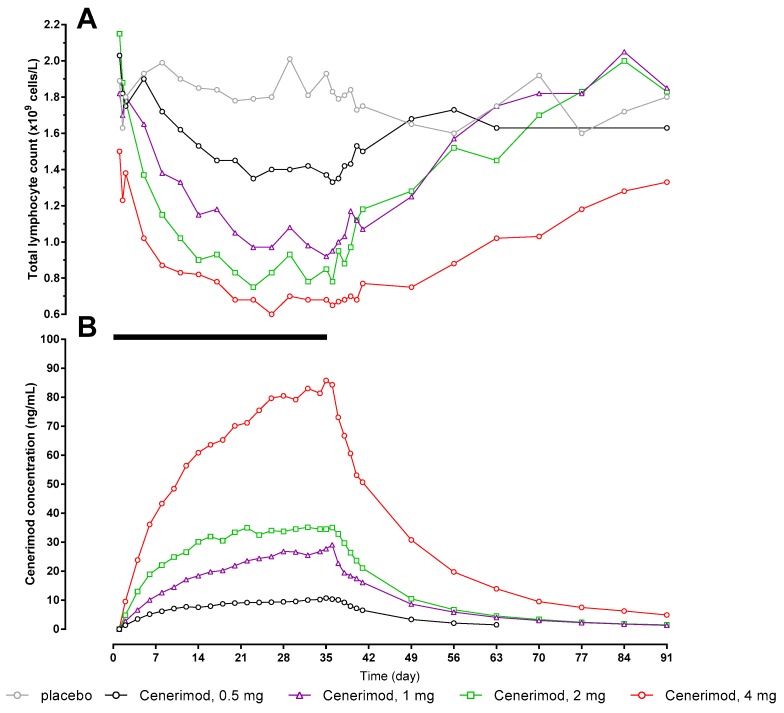
Arithmetic mean total lymphocyte count (Panel (**A**)) and cenerimod concentration (Panel (**B**)) versus time profiles from 0 h to EOS after multiple-dose o.d. oral doses of cenerimod (Study 2) for 35 days (*N* = 6 per dose group, *N* = 8 for placebo group). The black bar represents the treatment duration. EOS: end-of-study.

The PK/PD relationship has been investigated and is depicted in Figure 3. Based on Study 2 data, concentration and decrease in lymphocyte count vs. time follow similar profiles.

### 2.4. Safety and Tolerability

Apart from a severe and serious adverse event of circulatory collapse reported in Study 1 (6 h after administration of 25 mg cenerimod, lowest supine blood pressure (BP): 39/23 mmHg, pulse rate: 60 bpm, resolved without sequelae within 24 h following administration of atropine and ephedrine), all adverse events (AEs) were of mild-to-moderate intensity and resolved without sequelae. Most of the observed AEs were considered unrelated to study drug. The serious adverse event defined the maximum tolerated single dose of 10 mg. Pooling of the 3 studies revealed a similar number of subjects experiencing at least one AE following cenerimod (36 subjects, 64%) or placebo (11 subjects, 69%).

The most frequently reported AEs were headache, dizziness, chest pain (this combined chest pain, chest discomfort, musculoskeletal chest pain, and non-cardiac chest pain), and nasopharyngitis (Table 3). There were no apparent drug-related effects on body temperature, clinical chemistry variables, coagulation variables, or physical examination.

**Figure 4:**
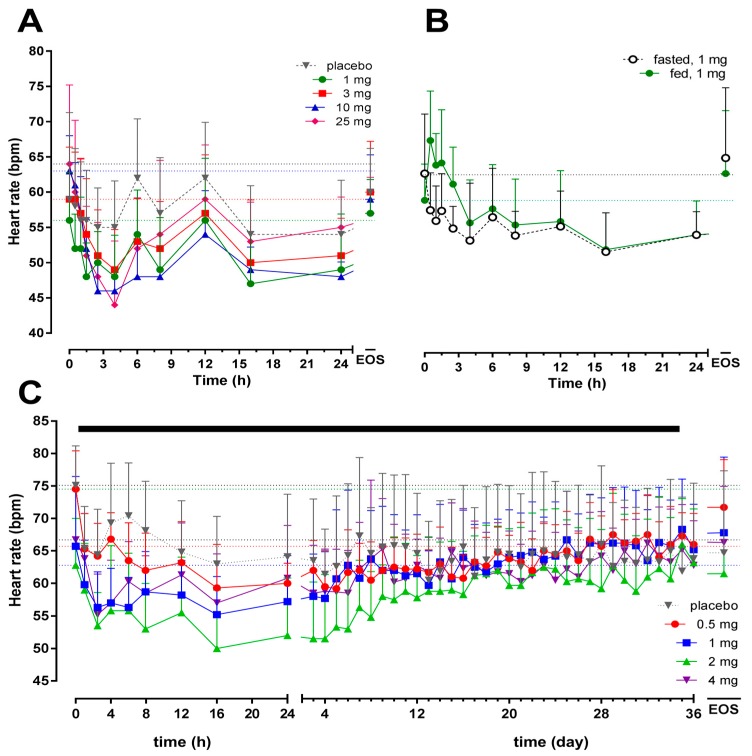
Arithmetic mean (with standard deviation) heart rate versus time profiles from 0 h to EOS after single-dose administration of cenerimod (Panel (**A**), *N* = 6 per dose group, *N* = 8 for placebo group), single-dose administration in fed or fasted conditions (Panel (**B**), *N* = 8), and after multiple o.d. oral doses for 35 days (Panel (**C**), *N* = 6 per dose group, *N* = 8 for placebo group). The black bar represents the treatment duration, left part effect over 24 h and right part over 36 days until EOS. The horizontal dotted lines represent the baseline values. EOS: end-of-study.

As expected, based on many other S1P_1_R modulators in development [15], a transient decrease in HR was observed following start of cenerimod administration. As shown in Figure 4A, this decrease was dose-dependent in Study 1. The maximum decrease from baseline (mean ± SD) was −8 ± 9 (*p* = 0.055 vs. baseline), −10 ± 5 (*p* = 0.03 vs. baseline), −17 ± 7 (*p* < 0.001 vs. baseline), and −21 ± 11 (*p* = 0.007 vs. baseline) bpm 4 h after cenerimod administration at doses of 1, 3, 10 and 25 mg, respectively. Placebo administration led to a slight decrease in HR (maximum change from baseline (mean ± SD): −4 ± 9; *p* = 0.43 vs. baseline). When compared to placebo, a significant decrease in HR was observed after single doses of 10 and 25 mg (*p* = 0.007). A dose–response relationship was not observed in Study 2 and the maximum mean ± SD change from baseline was −10 ± 4 (*p* = 0.0001 vs. baseline), −9 ± 6 (*p* = 0.004 vs. baseline), −9 ± 5 (*p* = 0.002 vs. baseline), and −12 ± 5 (*p* < 0.001 vs. baseline) bpm 2.5 h after the first administration of cenerimod at doses of 0.5, 1, 2 and 4 mg, respectively (Figure 4C). This effect was observed following the first dose and the second administration of cenerimod (Day 2) led to a less pronounced decrease in HR, which returned to baseline values within 7 to 14 days. Maximum change from baseline (mean ± SD) in the placebo group was −11 ± 8 bpm (*p* = 0.006 vs. baseline). In Study 3, no significant difference was observed between the change from baseline (mean ± SD) in fasted (−11 ± 10 bpm; *p* = 0.04 vs. baseline) and fed (−7 ± 5 bpm; *p* = 0.276 vs. baseline) conditions (*p* = 0.297 fed vs. fasted; Figure 4B).

## 3. Discussion

This study provides the first PK, PD, safety, and tolerability data for cenerimod, a selective oral S1P_1_R modulator, in healthy male subjects. Single and multiple o.d. oral doses of up to 10 and 4 mg (up to 35 days), respectively, were well tolerated. The nature and severity of AEs reported in the present studies were similar to those observed following administration of other representatives of this drug class, e.g., ponesimod [7,11,12], fingolimod [17,18], or siponimod [19]. Furthermore, decreases in BP were triggered following single high doses of cenerimod as previously observed with ponesimod [11].

Pharmacokinetic evaluation revealed that exposure to cenerimod was slightly more than dose-proportional for single doses and dose-proportional across the entire dose range for multiple doses. The intersubject variability was low to moderate for *C*_max_ (9.1–27.3%), AUC_0–24_ (11.0–27.6%), and *t*_1/2_ (5.3–28.6%). Multiple-dose PK of cenerimod are characterised by steady-state concentrations reached 20–32 days following o.d. administration and exposure is approximately 5- to 9-fold greater than after the initial dose. Compared to single-dose, multiple-dose PK revealed similar *t*_max_ (4–6 h), but longer, dose-dependent *t*_1/2_ (283–539 h), and higher intersubject variability (≤45.0%). These PK properties of cenerimod resemble those of fingolimod [20] or amiselimod [21] more than of ponesimod [11,12] or siponimod [19]. Based on the present data and its lipophilic properties, it is plausible that cenerimod accumulates in several tissues.

As expected, based on its pharmacological mode of action, cenerimod decreased the lymphocyte count in peripheral blood. Following single-dose administration, this reduction was different from placebo at doses ≥3 mg and was dose-dependent. Single- and multiple-dose administration of 25 and 2 mg led to a maximal decrease of circulating lymphocytes of 76% and 64%, respectively. This is in good agreement with previous data obtained from fingolimod and ponesimod administered at supra-therapeutic doses [11,12,13,17,18]. The effect was sustained and reversible within 7 and 40 days after single- and multiple-dose administration of cenerimod. In line with multiple-dose administration of ponesimod or fingolimod, lymphocyte counts decreased and reached a plateau [12,13,17]. Interestingly, the percent change from baseline and AUEC were slightly higher in the 2 mg compared to the 4 mg dose group. This may be explained by a lower mean baseline of lymphocyte count in the 4 mg group compared to the 2 mg group since the same threshold was reached (~0.6 × 10^9^ cells/L) as shown in Table 2. Experience with other selective S1P_1_R modulators such as siponimod and ponesimod has shown that a lymphocyte count reduction of 60–70% from baseline is associated with a plateau of efficacy for treatment of multiple sclerosis, while a 20–30% reduction in lymphocyte count is associated with minimal efficacy [6,19].

The overall pattern of reported AEs is similar to the one observed with the non-selective S1P receptor modulator fingolimod or the selective S1P_1_R modulators ceralifimod, ozanimod and ponesimod [11,12,17,18,22,23]. Nevertheless, incidence of AEs such as bradycardia and dyspnoea was lower with cenerimod compared to fingolimod [17,18] or ponesimod [11,12]. Following single doses of 10 and 25 mg and multiple doses of 1 mg, a slight decrease in FEV_1_ was observed. This effect might be related to agonism of S1P_1_R although mainly S1P_3_ receptors have been shown to mediate S1P effects on the lung [24].

Although the role of S1P_3_ receptors in S1P-induced HR effect has been described [8,10], another signalling might be involved based on the HR effect of highly selective S1P_1_R modulators [11,12,19]. Previous studies have revealed that both S1P_1_ and S1P_3_ agonism prior to receptor internalisation [8,10,25] lead to a reduction in HR via stimulation of the inward rectifier potassium current (I_K.ACh_) [26]. Internalisation of S1P_1_R and their desensitisation explain that treatment initiation triggers a transient decrease in HR [27].

Cenerimod displays advantages over fingolimod and ponesimod in terms of selectivity on the S1P_1_R. In addition, the long *t*_1/2_ of cenerimod, compared to ponesimod, will allow a built-in natural uptitration and a longer duration for drug holiday [15].

Single doses of cenerimod up to 10 mg were well tolerated and the tolerability profile of cenerimod is similar to that of ponesimod. In conclusion, the present promising results warrant further development of cenerimod in patients suffering from autoimmune disorders, e.g., systemic lupus erythematosus (NCT02472795).

## 4. Materials and Methods

### 4.1. Subjects

In these three sub-studies, healthy male subjects aged between 18 and 47 years were included. The health of the subjects was assessed at the screening visit, which included recording of the medical history, medications taken during the 3 months preceding the screening visit, a physical examination, measurement of body weight and height, clinical laboratory tests, recording of vital signs, and standard electrocardiogram (ECG). At screening, subjects had to have PR interval <200 ms, HR 55–90 bpm, systolic and diastolic blood pressure (BP) 100–150 and 50–90 mm Hg, respectively, (FEV_1_) and (FVC) >80% of the predicted value, and a normal total lymphocyte count (>10^9^ lymphocytes/L). Written informed consent was obtained from each individual participating in the study prior to any study procedure and after adequate explanation of the aims, methods, objectives and potential hazards of the studies. The single-ascending dose study (Study 1) was performed prior to and in a different centre than the multiple-ascending dose (Study 2) and the food effect study (Study 3). All protocols were approved by the Reading and Plymouth Independent Ethics Committees (UK) (date of approval of initial protocol from Institutional Review Board (IRB), 10 November 2010 for SAD, 20 April 2012 for MAD and food effect). These studies were performed according to good clinical practice and in accordance with the principles of the Declaration of Helsinki.

### 4.2. Study Design

This work was performed in two centres located in the UK. Study 1 and Study 2 were single-centre, randomised, double-blind, placebo-controlled, parallel-group single- and multiple-ascending oral dose studies, respectively. Study 3 was a two-way cross-over, open-label, food effect study. A total of 72 male subjects (32, 32 and 8 subjects in Study 1, Study 2 and Study 3, respectively) were enrolled, 8 subjects were assigned per dose group (ratio 3:1 for active:placebo in Study 1 and Study 2). Female subjects were excluded based on the potential teratogenic risk of cenerimod. Cenerimod (capsule formulation) was administered at a single dose of 1, 3, 10 or 25 mg in Study 1 and o.d. for 35 days at doses of 0.5, 1, 2 and 4 mg in Study 2. In Study 3, a single dose of 1 mg cenerimod was administered either in fed (i.e., a high-fat and high-calorie (as described in the Food and Drug Administration (FDA) guidance [28], breakfast 30 min before cenerimod administration) or in fasted conditions, with a washout period of at least 45 days between the study drug administrations. All treatments were administered in the morning (fasted condition in Study 1 and Study 2) with approximately 240 mL of water. Based on the ratio of the lowest-observed-adverse-effect-levels in the 4-week rat and dog toxicology studies and human equivalent doses at the planned starting dose of 1 mg, the safety margins were 33.8 and 116.8, respectively. PK and PD profiles after multiple oral o.d. doses were predicted from data obtained during Study 1 and supported the selection of doses in Study 2.

Subjects were admitted to the clinical centre on Day −1 and remained in the clinic until Day 7 (Study 1 and Study 3) or Day 41 (Study 2) when they were discharged if this was allowed on the basis of their medical condition (HR ≥45 bpm or ≥70% of baseline, no clinically relevant treatment-emergent ECG abnormalities, FEV_1_ and FVC ≥80% of baseline, and absolute lymphocyte count ≥10^9^ lymphocytes/L). Subjects returned to the clinic for weekly outpatient visits (until Day 21 in Study 3, and in the 10 and 25 mg dose groups in Study 1, Day 56 for the 0.5 mg dose group in Study 2, and Day 84 in the 1, 2 and 4 mg dose groups in Study 2). An end-of-study visit comprising the same examinations as at the screening visit was conducted at least 28 days after the last administration of cenerimod.

### 4.3. Pharmacokinetic Assessments

In Study 1 and Study 3, blood samples of about 3 mL were collected in ethylene di-amine tetra acetic acid (EDTA) tubes pre-dose and at 0.5, 1, 1.5, 2.5, 4, 6, 8, 12, 16, 24, 36, 48, 72, 96, 120 and 144 h after study drug administration. In addition, blood PK samplings were performed on Day 14, Day 21 and Day 28 in Study 3, and in the 10 and 25 mg dose group in Study 1. In Study 2, blood sampling was performed pre-dose and 1, 2.5, 4, 6, 8, 12, 16 h on Day 1 and Day 35 for a full PK profile after the first and the last dose. Samples were collected pre-dose from Day 2 to Day 34 and also at each outpatient visit until the end-of-study examination. After centrifugation, plasma was transferred into a polypropylene tube and stored at −21 °C (±5 °C) pending analysis. Plasma concentrations of cenerimod were determined using a validated liquid chromatography coupled to tandem mass spectrometry assay with a lower limit of quantification of 0.1 ng/mL and using a pentadeuterated form of cenerimod as internal standard. The method was linear in the concentration range 0.1–100 ng/mL. Analysis of quality-control samples of all runs showed that inter-batch coefficients of variation (precision) were <8.8%, whereas the average intra-batch accuracy was in the range 96.0–102.7%. Noncompartmental PK analyses were performed using Professional WinNonlin 6.1 software (Pharsight Corp., Mountain View, CA, USA). The variables *C*_max_ and *t*_max_ were directly obtained from the plasma concentration-time profiles, AUC_0–t_ was calculated using the trapezoidal method [29], and *t*_1/2_ was calculated as ln 2/λz, where λz is the terminal elimination rate constant estimated by log-linear regression analysis.

### 4.4. Pharmacodynamic Assessments

Analysis of the lymphocyte count in peripheral blood was performed at the same time points as PK samples in Study 1 and Study 3. In Study 2, lymphocyte count was assessed on Day 1, every 3 days from Day 2 to Day 35, and at each outpatient visit. Assessment of lymphocyte count was part of the clinical haematology evaluation. To assess lymphocyte count, blood samples of 2.7 mL were collected into a K_3_-EDTA polypropylene tube and analysis was performed using a cell counter. The measured individual whole blood lymphocyte counts were used to establish the effect-time curve, lymphocyte nadir (*E*_max_), and time to nadir (*t*_max_). The area under the effect-curve (AUEC) was calculated according to the linear trapezoidal rule using the measured lymphocyte count (effect)-time values.

### 4.5. Safety and Tolerability Assessments

Safety and tolerability were evaluated by monitoring AEs, vital signs measurements (supine BP), 12-lead ECG recordings, pulmonary function tests (PFTs), clinical laboratory, physical, and neurological examinations. ECG variables and BP were recorded pre-dose and 0.5, 1, 1.5, 2.5, 4, 6, 8, 12, 16, 24, 36, 48, 72, 96, 120 and 144 h after study drug administration in Study 1. For the single-dose groups of 10 and 25 mg in Study 1 and for Study 3, study day 14, 21 and 28 time points were added. In Study 2, recordings of BP and 12-lead ECG were performed pre-dose and 1, 2.5, 4, 6, 8, 12, 16 h post-dose on Day 1 and pre-dose and 4, 6, 8, 12 h post-dose from Day 2 to Day 35. Additional recordings were done at each outpatient visit.

### 4.6. Statistical Analysis

Pharmacokinetic variables were analysed descriptively providing the geometric mean and 95% CIs for *C*_max_, AUC_0–24_, AUC_0–∞_, and *t*_1/2_, and the median with the range for *t*_max_. Dose proportionality of log transformed *C*_max_, AUC_0–24_, and AUC_0–∞_ values was explored by the power model [30]. Pharmacodynamic and cardiodynamic data are expressed as mean ± SD. The Student’s *t*-test was used to analyse the effects of cenerimod on the lymphocyte count and HR. Comparisons of baseline versus nadir (i.e., lowest value measured) and placebo vs. treatment were performed. Safety and tolerability data were analysed descriptively by treatment group and data from placebo subjects were pooled. Differences were considered to be statistically significant at *p* < 0.05.

SAS^®^ software, version 9.2 (SAS Institute, Cary, NC, USA) was used for the statistical analysis and descriptive statistics of clinical and PK data.

## Figures and Tables

**Table 1:** Pharmacokinetic parameters of cenerimod estimated following single-dose (Study 1), o.d. for 35 days (Study 2), and single-dose administration in fed or fasted conditions (Study 3).

		Day 1					Day 35						
	Dose (mg)	*C*_max_ (ng/mL)	*t*_max_ (h)	AUC_0–24_ (ng·h/mL)	AUC_0__–∞_ (ng·h/mL)	*t*_1/2_ (h)	*C*_max_ (ng/mL)	*t*_max_ (h)	AUC_0–24_ (ng·h/mL)	AUC_0__–∞_ (ng·h/mL)	*t*_1/2_ (h)	R	
												*C*_max_	AUC_0–24_
Study 1	1	3.9	5.0	69.3	610	172	NC						
		(2.9–5.2)	(4.0–8.0)	(52.4–90.3)	(504–735)	(125–232)							
	3	13.1	6.0	232	2070	184							
		(10.1–16.8)	(4.0–8.0)	(181–292)	(1470–2790)	(153–220)							
	10	46.7	6.0	823	8030	199							
		(42.4–51.3)	(6.0–8.0)	(732–923)	(6700–9550)	(188–211)							
	25	122	6.2	2030	18,700	170							
		(89.5–161.0)	(4.0–8.0)	(1480–2700)	(14,100–24,200)	(134–213)							
Study 2	0.5	2.4	5.0	41.1	NC	NC	13.1	4.3	269	3390	283	5.5	6.6
		(2.0–2.8)	(4.0–6.0)	(37.0–45.5)			(9.8–17.6)	(2.5–8.0)	(198–366)	(2050–5590)	(210–381)	(4.6–6.5)	(5.3–8.1)
	1	5.1	5.0	83.5			34.2	5.0	720	9750	436	6.7	8.6
		(4.4–5.9)	(4.0–6.0)	(72.9–95.6)			(26.8–43.7)	(2.5–8.0)	(560–926)	(7270–13,100)	(359–529)	(5.5, 8.3)	(7.3, 10.2)
	2	7.7	6.0	134			43.9	6.0	925	11,900	415	5.7	6.9
		(6.1–9.7)	(4.0–8.0)	(109–164)			(35.9–53.8)	(6.0–8.0)	(767–1120)	(8340–16,900)	(358–481)	(4.3, 7.5)	(5.2, 9.3)
	4	18.9	5.0	301			98.7	6.0	2100	31,900	539	5.2	7
		(14.4–24.9)	(4.0–6.0)	(238–380)			(68.0–143.0)	(2.5–12.0)	(1390–3170)	(21,200–48,000)	(492–591)	(4.4, 6.3)	(5.6, 8.7)
Study 3	1 fasted	4.73	6.0	83.7	649	200	NC						
		(4.1, 5.5)	(6.0–8.0)	(71.9, 97.4)	(507, 832)	(166, 239)							
	1 fed	4.35	7.0	75.5	630	191							
		(3.5, 5.4)	(6.0–12.0)	(62.0, 91.9)	(498, 798)	(157, 232)							

The data are expressed as geometric mean (95% confidence interval), execpt *t*_max_ that is expressed as median (range). AUC_0–∞_: area under the curve from time zero to infinity, AUC_0–24_: area under the curve from time zero to 24 h, *C*_max_: maximum plasma concentration, NC: not calculated, R ratio Day35/Day1, *t*_1/2_: terminal half-life, *t*_max_: time to maximum plasma concentration.

**Table 2:** Pharmacodynamic variables estimated following single-dose (Study 1), o.d. for 35 days (Study 2), and single-dose administration in fed or fasted conditions (Study 3).

	Dose (mg)	*t*_max_ (h)	*E*_max_ (×10^9^/L)	AUEC (h·10^9^/L)			*t*_max_ (day)	*E*_max_ (×10^9^/L)	AUEC (day·10^9^/L)	*t*_max_ (day)	*E*_max_ (×10^9^/L)	AUEC (day·10^9^/L)
		Day 1					Day 1 to Day 36			Day 36 to EOS		
Study 1	1	39.1	1.9	358	Study 2	0.5	28.2	1.2	54	36.5	1.3	44
		(58.1)	(0.5)	(60)			(4.4)	(0.1)	(5)	(0.5)	(0.2)	(4)
	3	13.7	1.3	1185		1	27.2	0.9	42	36.2	0.9	88
		(16.8)	(0.2)	(232)			(6.7)	(0.2)	(11)	(0.4)	(0.2)	(9)
	10	7.7	0.6	801		2	25.2	0.6	35	36.7	0.8	85
		(2.3)	(0.2)	(188)			(7.3)	(0.2)	(5)	(1)	(0.1)	(22)
	25	8	0.4	799		4	21	0.6	28.1	37.7	0.6	55
		(4)	(0.2)	(254)			(3.1)	(0.1)	(2)	(2)	(0.1)	(9)
	placebo	66.5	1.5	1110		placebo	9.3	1.5	65.2	63.2	1.3	93
		(106.8)	(0.3)	(615)			(12.5)	(0.3)	(14.3)	(19)	(0.2)	(22)
Study 3	1 fasted	3.8	1.5	46								
		(2.5)	(0.3)	(8)								
	1 fed	3.1	1.4	47								
		(4)	(0.4)	(8)								

The data are expressed as mean (SD). AUEC: area under the effect-time curve, *t*_max_: time to nadir (minimum lymphocyte count), and *E*_max_: nadir value in lymphocyte count (×10^9^ cells/L).

**Table 3:** Treatment-emergent adverse events (AEs) in Study 1, Study 2 and Study 3.

	Study 1					Study 2					Study 3	
Treatment	1 mg	3 mg	10 mg	25 mg	placebo	0.5 mg	1 mg	2 mg	4 mg	placebo	fasted	fed
Number of subjects dosed	6	6	6	6	8	6	6	6	6	8	8	8
Number of subjects with at least one AE (%)	1 (17)	6 (100)	1 (17)	4 (67)	3 (38)	4 (67)	5 (83)	6 (100)	4 (66.7)	8 (100)	4 (50)	3 (38)
Number of subjects reporting an event (%)												
Constipation	-	4	-	-	-	-	-	-	-	-	-	-
Headache	-	-	-	3	1	2	2	2	3	1	1	1
Dizziness	-	1	1	-	1	-	1	1	2	-		
Presyncope	-	-	-	2	-	-	-	-	-	-	-	-
Bradycardia	-	-	-	1	-	-	-	-	-	-	-	-
Circulatory collapse	-	-	-	1 *	-	-	-	-	-	-	-	-
Diarrhoea	-	-	-	1	-	-	1	-	1	-		
Nausea	-	-	-	1	-	-	-	-	-	3		
Pain in extremity	-	-	-	-	1	-	-	-	-	-	-	-
Paraesthesia	-	1	-	-	-	-	-	-	-	-	-	-
Rhinitis	-	1	-	-	-	-	-	-	-	-	-	-
Seborrheic dermatitis	-	-	-	-	1	-	-	-	-	-	-	-
Syncope	-	-	1	-	-	-	-	-	-	-	-	-
Upper respiratory tract infection	1	-	-	-	-	-	-	-	-	-	-	-
Chest pain	-	-	-	-	-	1	2	2	3	2	2	-
Nasopharyngitis	-	-	-	-	-	-	1	1	2	3	2	1
Abdominal pain	-	-	-	-	-	-	1	1	-	1	-	-
Back pain	-	-	-	-	-	-	-	2	1	-	-	-
Neck pain	-	-	-	-	-	-	1	1	-	1	-	-
Dyspepsia	-	-	-	-	-	-	1	1	-	-	-	-
Joint injury	-	-	-	-	-	-	-	1	-	1	-	-
Oropharyngeal pain	-	-	-	-	-	-	1	1	-	-	-	1
Excoriation	-	-	-	-	-	-	-	-	-	-	1	-
Nasal congestion	-	-	-	-	-	-	-	-	-	-	-	1
Rash	-	-	-	-	-	-	-	-	-	-	1	-
Dyspnoea	-	-	-	-	-	-	-	1	-	-	-	-

* Serious adverse event.

## Abbreviations

AE	adverse event
AUC_0–24_	area under the plasma drug concentration-time curve from 0 to 24 h
AUC_0–∞_	area under the plasma drug concentration-time curve from 0 to infinity
AUC	area under the plasma drug concentration-time curve
AUEC	area under the effect-time curve
CI	confidence interval
CL/F	apparent oral clearance
*C*_max_	maximum plasma concentration
*E*_max_	maximum effect
ECG	electrocardiogram
EDTA	ethylene di-amine tetra acetic acid
FEV_1_	forced expiratory volume in 1 s
FVC	forced vital capacity
HR	heart rate
LS	least squares
o.d.	once daily
PD	pharmacodynamics
PK	pharmacokinetics
S1P	sphingosine-1-phosphate
S1P_1_R	S1P_1_ receptor
SD	standard deviation
*t*_1/2_	terminal half-life
*t*_max_	time to reach maximum concentration
Vss/F	apparent volume of distribution at steady-state
